# Kidney health for all: bridging the gap in kidney health education and literacy

**DOI:** 10.1590/1414-431X2022e12161

**Published:** 2022-05-16

**Authors:** R.G. Langham, K. Kalantar-Zadeh, A. Bonner, A. Balducci, L.L. Hsiao, L.A. Kumaraswami, P. Laffin, V. Liakopoulos, G. Saadi, E. Tantisattamo, I. Ulasi, S.F. Lui, the World Kidney Day Joint Steering Committee©

**Affiliations:** 1 St. Vincent's Hospital, Department of Medicine, University of Melbourne, Melbourne, Victoria, Australia; 2 Division of Nephrology, Hypertension and Kidney Transplantation, Department of Medicine, University of California Irvine School of Medicine, Orange, CA, USA; 3 School of Nursing and Midwifery, Griffith University, Southport, Queensland, Australia; 4 Italian Kidney Foundation, Rome, Italy; 5 Brigham and Women's Hospital, Renal Division, Department of Medicine, Boston, MA, USA; 6 Tamilnad Kidney Research (TANKER) Foundation, The International Federation of Kidney Foundations - World Kidney Alliance (IFKF - WKA), Chennai, India; 7 International Society of Nephrology, Brussels, Belgium; 8 Division of Nephrology and Hypertension, 1st Department of Internal Medicine, AHEPA Hospital, Aristotle University of Thessaloniki, Thessaloniki, Greece; 9 Nephrology Unit, Department of Internal Medicine, Faculty of Medicine, Cairo University, Giza, Egypt; 10 Renal Unit, Department of Medicine, College of Medicine, University of Nigeria, Ituku-Ozalla, Enugu, Nigeria; 11 International Federation of Kidney Foundations - World Kidney Alliance, The Jockey Club School of Public Health and Primary Care, The Chinese University of Hong Kong, Hong Kong, China

**Keywords:** Educational gap, Health literacy, Health policy, Information technology, Kidney health, Social media

## Abstract

The high burden of kidney disease, global disparities in kidney care, and the poor outcomes of kidney failure place a growing burden on affected individuals and their families, caregivers, and the community at large. Health literacy is the degree to which individuals and organizations have, or equitably enable individuals to have, the ability to find, understand, and use information and services to make informed health-related decisions and actions for themselves and others. Rather than viewing health literacy as a patient deficit, improving health literacy lies primarily with health care providers communicating and educating effectively in codesigned partnership with those with kidney disease. For kidney policy makers, health literacy is a prerequisite for organizations to transition to a culture that places the person at the center of health care. The growing capability of and access to technology provides new opportunities to enhance education and awareness of kidney disease for all stakeholders. Advances in telecommunication, including social media platforms, can be leveraged to enhance persons’ and providers’ education. The World Kidney Day declares 2022 as the year of “Kidney Health for All” to promote global teamwork in advancing strategies in bridging the gap in kidney health education and literacy. Kidney organizations should work toward shifting the patient-deficit health literacy narrative to that of being the responsibility of health care providers and health policy makers. By engaging in and supporting kidney health-centered policy making, community health planning, and health literacy approaches for all, the kidney communities strive to prevent kidney diseases and enable living well with kidney disease.

## Introduction

Given the high burden of kidney disease and global disparities related to kidney care, in carrying forward our mission of advocating *Kidney Health for All*, the challenging issue of bridging the well-identified gap in the global understanding of kidney disease and its health literacy is the theme for World Kidney Day (WKD) 2022. Health literacy is defined as the degree to which people and organizations have - or equitably enable individuals to have - the ability to find, understand, and use information and services to make informed health-related decisions and actions for themselves and others (1). Not only is it increasingly recognized that health literacy plays a critical role in outcomes for individuals with kidney disease and the community in general, but it is also increasingly important that policy makers worldwide are informed and aware of the opportunities and actual measurable outcomes that can be achieved through kidney-specific preventative strategies.

## The global community of people with kidney disease

Most people are not aware what their kidneys are for or where their kidneys are even located. For those affected by a disease and the subsequent effects on overall health, effective communication with the health care provider is required to help them understand what to do, make decisions, and take action. Health literacy involves more than the functional abilities of an individual; it also involves the cognitive and social skills needed to access, understand, and use information to manage health conditions (2). It is also contextual (3), because as health needs change, so does the level of understanding and ability to solve problems. Health literacy is, therefore, an interaction between individuals, health care providers, and health policy makers (4). This is why the imperatives around health literacy are now recognized as indicators for quality of local and national health care systems and health care professionals within it (5). As chronic kidney disease (CKD) progresses alongside other health changes and increasing treatment complexities, it becomes increasingly difficult for individuals to manage (6). Health policy has been promoting care partnerships between health-centered policy, community health planning, and health literacy for about a decade (7), but current approaches need to evolve (Table 1).

**Table 1:** Summary of kidney health promotion strategies, involving kidney health policy, community kidney health planning, and kidney health literacy, and proposed future direction.

Kidney health promotion	Definition	Stakeholders	Current status	Limitations/challenges	Suggested solutions/future research
Kidney health- centered policy	Incorporate kidney health into policy decision makingPrioritize policies with primary prevention for CKD	GovernancePolicy makersInsurance agencies	Policy emphasizing treatment for CKD and kidney failure rather than kidney health prevention	Economic-driven situation challenging CKD risk factor minimization (e.g., food policy)	Promote implementation of public health program for primary CKD preventionPromote sustainable treatment for CKD and dialysisIncrease kidney transplant awarenessEnhance visibility and encourage brother-sister nephrology and transplant program in LMICSupport research funding from governmentHealth care cost-effectiveness for caring for CKDKidney failure, including maintenance dialysis and transplantPromote surveillance programs for kidney diseases and their risk factors
Community kidney health planning	Building up preventive strategies to promote healthy communities and primary health care facilities	Community leadershipKidney patient advocacy	Belief in community leaders in LMIC	Education and understanding kidney health promotion of community leadership and people	Improve role model of communityEnhance kidney support networks
Kidney health literacy	Receive knowledge, skills, and information on how to be healthy	People with CKDCare partnersHealth care providers	Lack of awareness of CKD and risk factorsCare partner burden and burnoutInadequate health care workersHigh patients-to-health care workers ratio, especially in rural areas	Inadequate policy directionIneffective communication skills of health care providers	Organizational paradigm shift toward health literacyImproving communication between health care providers with patients and care partnersUsing teach-back methods for consumer educationAdapting technologies for appropriate health literacy and sociocultural environmentsFamily engagement in patient careIncentive for community health care providers in rural areas

CKD: chronic kidney disease; LMIC: low- to middle-income country.

Assessing health literacy requires appropriate multidimensional patient-reported measures, such as the World Health Organization-recommended Health Literacy Questionnaire (available in over 30 languages) rather than tools measuring only functional health literacy (e.g., Rapid Estimate of Adult Literacy in Medicine or Short Test of Functional Health Literacy in Adults) (8). It is therefore not surprising that studies have demonstrated that low health literacy (LHL) in people with CKD is associated with poor CKD knowledge, self-management behaviors, and health-related quality of life, and greater comorbidity severity (7). Unfortunately, most CKD studies have measured only functional health literacy, so the evidence that LHL results in poorer outcomes, especially higher health care utilization and mortality (9), and reduces access to transplantation (10), is weak.

Recently, health literacy has been considered to be an important bridge between lower socioeconomic status and other social determinants of health (4). Indeed, this is not a feature that can be measured by the gross domestic product of a country, as the effects of LHL on the extent of CKD in the community are experienced globally, regardless of country income status. The lack of awareness of kidney disease risk factors, even in those with high health literacy abilities, is testament to the difficulties in understanding this disease and why the United States, for instance, recommends that a universal precaution approach toward health literacy is undertaken (11).

So, what does the perfect health literacy program look like for people with CKD? In several high-income countries, there are national health literacy action plans with the emphasis shifted to policy directives, organizational culture, and health care providers. In Australia, for instance, a compulsory health literacy accreditation standard makes the health care organization responsible for ensuring that providers are cognizant of individual health literacy abilities (12). Many high-income countries, health care organizations, nongovernmental organizations, and jurisdictions are providing an array of consumer-focused web-based programs that provide detailed information and self-care training opportunities. However, most programs are largely designed for individual/family use that are unlikely to mitigate LHL. There is, however, substantial evidence that interventions improving health care provider communication are more likely to improve understanding of health problems and abilities to adhere to complex treatment regimens (13).

The goal is to provide access to information that is authentic and tailored specifically to individual and community needs. The challenge is particularly evident in more remote and low- to middle-income countries, specifically the importance of culturally appropriate knowledge provision. The principals for improving health literacy are the same, but it is important to understand how to proceed and to empower consumers, with a codesign approach, which is critical and may result in a different outcome in more remote parts of the world. This principal is especially true in smaller communities, with less access to electronic communication and health care services, where the level of health literacy is shared across the community and where what affects the individual affects the entire community. Decision support systems are different, led by elders, and in turn, educational resources are best aimed at improving knowledge of the whole community.

A systematic review of the evaluation of interventions and strategies shows that this area of research is still at an early stage (14), with no studies unravelling the link between LHL and poor CKD outcomes. The best evidence is for targeted programs to improve the communication skills of health care professionals. A good example is Teach-back, a cyclical, simple, and low-cost education intervention that shows promise for improving communication, knowledge, and self-management in the CKD population in low- and high-income countries (15). Furthermore, the consumer voice has articulated research priorities that closely align with principals considered important for educational success: building new educational resources, developed in partnership with consumers and focused on the needs of vulnerable groups. Indeed, programs that address the lack of culturally safe, person-centered, and holistic care and improve the communication skills of health professionals, are crucial for people with CKD (16).

## The networked community of kidney health care workers

**Figure 1:**
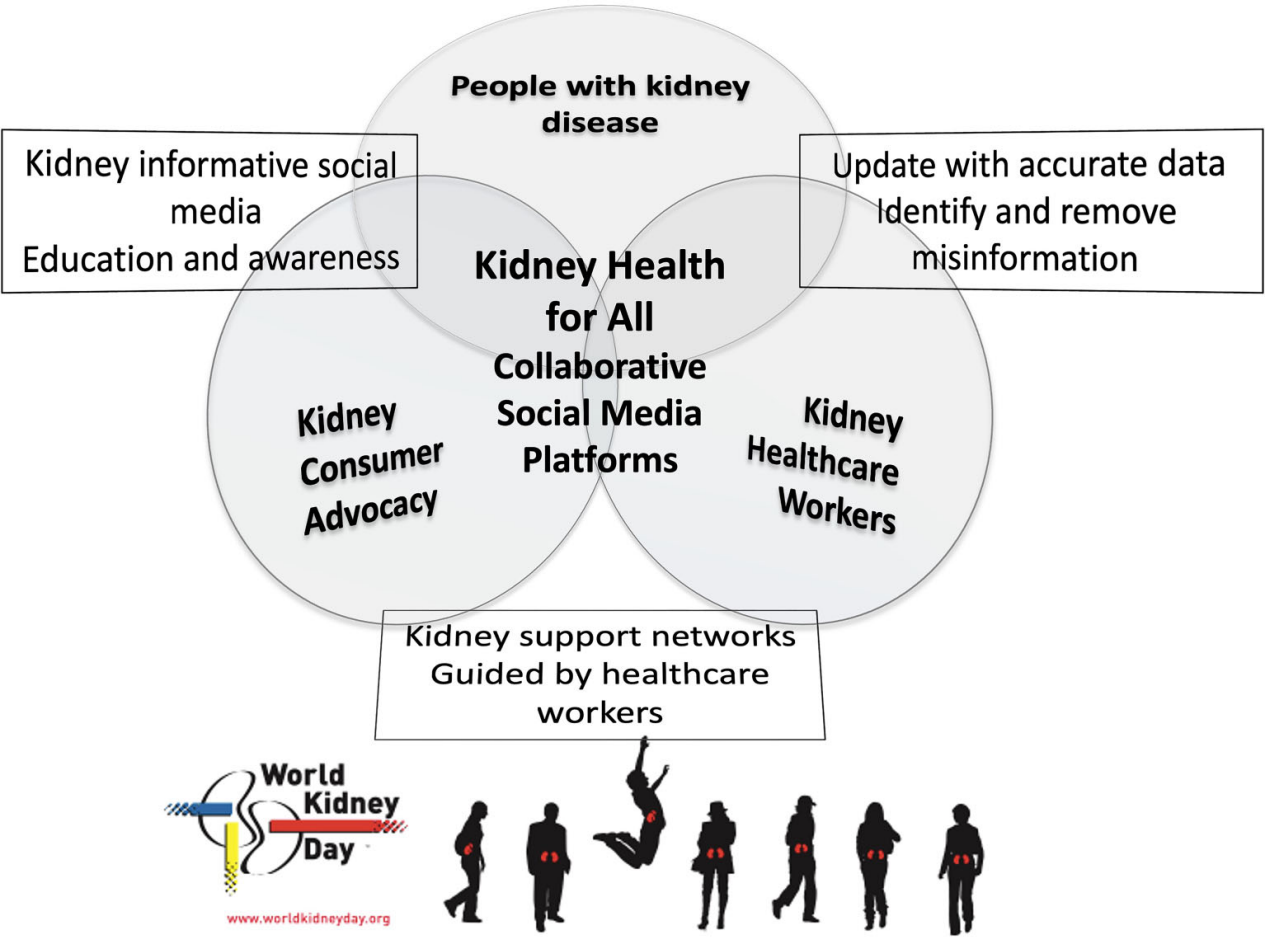
Schematic representation of consumer and health care professionals' collaborative advocacy using social media platforms with the goal of *Kidney Health for All*.

Non-physician health care workers, including nurses, advanced practice providers (physician assistants and nurse practitioners), and dietitians, pharmacists, social workers, technicians, physical therapists, and other health professionals, often spend more time with persons with kidney disease than nephrologists and other specialists. In the outpatient setting, at an appointment, in the emergency department, or in the inpatient setting, these health care professionals often see and interact with the patient first, last, and in between, as the encounters with the physician are often short and focused. Hence, the non-physician health care workers have many opportunities to talk with and empower individuals and their care partners about kidney disease issues (17,18). For instance, medical assistants can help identify people with CKD or at risk of developing CKD and educate them and their family members about the role of diet and lifestyle changes in the primary, secondary, and tertiary prevention of CKD while waiting to see the physician (19). Some health care workers provide connections to kidney patient advocacy groups and kidney support networks, which have been initiated or expanded via social media platforms (Figure 1) (20,21). Studies examining the efficacy of social media in kidney care and advocacy are underway (22,23).

Like physicians, many activities of non-physician health care workers have been increasingly affected by the rise of electronic health records and growing access to internet-based resources, including social media, that offer educational materials on kidney health, including kidney-preserving therapies with traditional and new interventions (24). These resources can be used for self-education and networking and promoting kidney disease awareness and learning. An increasing number of health care professionals are engaging in some types of social media activities, as shown in Table 2. At the time of this writing, Facebook, Instagram, Twitter, LinkedIn, and YouTube are the leading social media used by many - but not all - kidney health care workers. In some regions of the world, certain social media are used more frequently than others because of unique cultural circumstances or access (e.g., WeChat is a platform often used by health care workers and patient groups in China). Some health care professionals, such as managers and those in leadership and advocacy positions, may choose to use social media to engage people with CKD and their care partners or other health care professionals in alliance building and marketing. To this end, effective communication strategies and skills to use social media responsibly can provide clear advantages, as these skills and strategies are different for people with LHL and may need to be adapted. It is imperative that health care providers receive knowledge and training for a responsible approach to social media, so that these strategies are utilized with the needed awareness of their unique strengths and pitfalls (25): i) the confidentiality of consumers and care partners must not be violated when something is posted on social media, even by indirectly referring to a specific individual or a describing a specific condition unique to a specific person (e.g., when asking for transplant kidney donors on social media) (26,27); ii) confidential information about clinics, hospitals, dialysis centers, or similar health care and advocacy entities may not be disclosed on social media without ensuring that the needed processes, including obtaining permission to disclose, are followed; iii) the jobs and careers of health care workers should remain protected with thorough review of the content of the messages and illustrations/videos before posting online; iv) careless and disrespectful language and emotional tones are often counterproductive and may not be justified under the context of freedom of speech.

**Table 2:** Social media that are more frequently used for kidney education and advocacy.

Social media	Strength	Limitations	Additional comments
Facebook	Social media platform frequently used by many kidney patients and patient groups	Widely used for entertaining purposes, which can dilute its professional utility	User-friendly platform for kidney advocacy, enabling wide ranges of outreach goals
Instagram	Photo-based platform	Not frequently used by health care professionals	Picture friendly, potentially effective for illustrative educational purposes
Twitter	Often used by physician specialists and scientists, including nephrologists	Less frequently used by patients and care partners	Increasing popularity among physician and specialty circles
LinkedIn	More often used by professionals, including in industry	Originally designed for employment and job-seeking networking	Mostly effective to reach out to industry and managerial professionals
YouTube	Video-based platform	Less effective with non-video-based formats	Wide ranges of outreach and educational targets
WeChat	Widely used in mainland China	Access is often limited to those living in China or its diaspora	Effective platform to reach out to patients and health care professionals in China
Pinterest	Picture-based, often used by dietitians	Currently limited use by some health care workers	Useful for dietary and lifestyle education

Other popular social media at the time of this publication include, but not limited to, Tik Tok, Snapchat, Reddit, Tumblr, Telegram, Quora, and many others that are currently only occasionally used in kidney advocacy activities. Mobile and social media messaging apps include, but not limited to, WhatsApp, Zoom, Facebook Messengers, Skype Teams, and Slack. Platforms that are more often used as internet-based messaging are not included.

## The global kidney community of policy and advocacy

Policy and advocacy are recognized tools that, when used properly, can lead to change and paradigm shift at the jurisdictional level. Advocating for policy change to better address kidney disease is in itself an exercise in improving the health literacy of policy makers. Policy development, at its core, is about a key stakeholder or stakeholder group (e.g., kidney community that believes that a problem exists that should be tackled through government action). There is an increasing recognition of the importance of formulating succinct, meaningful, and authentic information, to improve health literacy and call governments to action.

Robust and effective policy is always underpinned by succinct and applicable information; however, the development and communication of this information, designed to bridge the knowledge gap of relevant jurisdictions, is only part of the policy development process. An awareness of the process is important for clinicians who want to advocate for effective change in prevention or improvement of outcomes in the CKD community.

**Figure 2:**
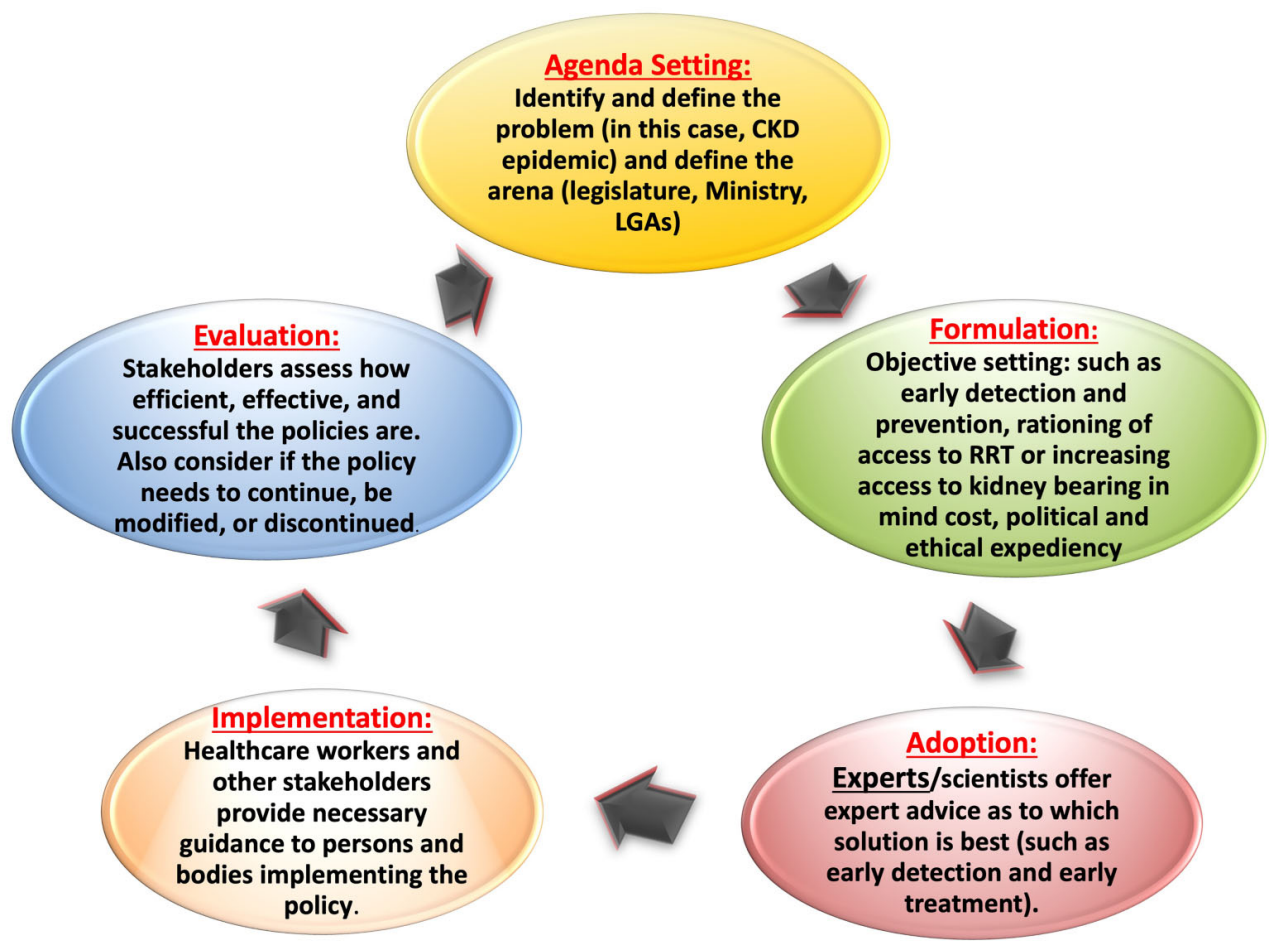
Policy cycle involving 5 stages of policy development. CKD: chronic kidney disease; RRT: renal replacement therapy; LGA: local government area.

Public policies, the plans for future action adopted by governments, are formulated through a political process in response to stakeholder observation and are usually written as a directive, law, regulation, procedure, or circular. Policies are purposely fit and directed toward specific goals and societal problems and usually consist of a chain of actions taken to solve those societal problems (28). Policies are an important output of political systems. Policy development can be formal and go through rigorous lengthy processes before adoption (such as regulations), or it can be less formal and adopted quickly (such as circulars). As already mentioned, government action envisioned by key stakeholders as a solution to a problem is at the core of policies. The process allows stakeholders to express their views and voice their concerns. Authentic information that is meaningful to the government is critical. The policy development process can be stratified into 5 stages (i.e., the policy cycle) as presented by Anderson in 1994 (29) and adapted and modified by other authors (30) (Figure 2). The policy cycle constitutes an expedient framework for evaluating the key components of the process.

The policy then moves to the implementation phase. This phase may require subsidiary policy development and adoption of new regulations or budgets (implementation). Policy evaluation is an essential part of policy processes and applies evaluation principles and methods to assess the content, implementation, or impact of a policy. Evaluation facilitates understanding and appreciation of the value and merits of a policy and the need for improvement. Of the 5 principles of advocacy that underlie policy making (31), the most important for clinicians engaged in this area is that of commitment, persistence, and patience. Advocacy takes time to yield the desired results.

The Advocacy Planning Framework, developed by Young et al. (30), consists of overlapping circles representing 3 sets of concepts (way into the process, the messenger, and the message and activities) that are key to planning any advocacy campaign: i) “Way into the process”: discusses the best approaches to translate ideas into the target policy debate and identify the appropriate target audience; ii) messenger: addresses the image maker or face of the campaign and other support paraphernalia that are needed; iii) message and activities: describe what can be said to key target audiences that is engaging and convincing and how best it can be communicated through appropriate communication tools.

Advocacy is defined as “an effort or campaign with a structured and sequenced plan of action, which starts, directs, or prevents a specific policy change” (31). The goal is to influence decision makers by communicating directly with them or getting their commitment through secondary audiences (advisers, the media, or the public) so that the decision maker understands the ideas, is convinced, takes ownership of the ideas, and is ultimately compelled to act (31). As with improving health literacy, communicating the ideas to policy makers is critical to ensuring policy implementation. There is much to be done to bridging this gap in understanding the magnitude of the burden of CKD in the community. Without good communication, many good ideas and solutions will not reach the communities and countries where they are needed. Therefore, aligned with the principles of developing resources for health literacy, the approach must be tailored to local needs.

Advocacy requires gaining momentum and support for the proposed policy or recommendation. The process is understandably slow, as it involves discussions and negotiations for paradigms, attitudes, and positions. In contemplating advocacy activities, multiple factors must be considered, which, interestingly, are not too different from those of building health literacy resources: What barriers stand in the way of policy-making processes? What resources are available to enable the process to succeed? Is the policy objective achievable considering all variables? Is the identified problem already being considered by policy makers (government or multinational organizations)? Is there an interest or momentum for it? Understandably, if there is some level of interest and the government is already looking at the issue, it is likely that it will succeed.

Approaches include the following (31,32):

- Advising (researchers are commissioned to produce new evidence-based proposals to assist the organization in decision making).
- Activism: involves petitions, public demonstrations, posters, fliers, and leaflet dissemination, often used by organizations to promote a certain set of values.
- Media campaign: having public pressure on decision makers helps in achieving results.
- Lobbying: entails face-to-face meetings with decision makers; often used by business organizations to achieve their purpose.

Here lies the importance of effective and successful advocacy with stakeholders, including policy makers, health care professionals, communities, and key change makers in society. The WKD has aimed to play this role from the beginning. WKD has gained the trust of people by delivering relevant and accurate messages and supporting local leaders. It is celebrated by kidney care professionals, celebrities, people with the disease, and their caregivers around the world. To achieve the goal, a sustainable and successful implementation framework includes creativity, collaboration, and communication.

The ongoing challenge for the International Society of Nephrology and International Federation of Kidney Foundations - World Kidney Alliance, through the Joint Steering Committee of WKD, is to operationalize how to collate key insights from research and analysis to effectively feed the policy-making process at the local, national, and international levels and inform or guide decision making (i.e., increasing engagement of governments and organizations, such as the World Health Organization, United Nations, and regional organizations, especially in low-resource settings). There is a clear need for ongoing renewal of strategies to close the gap in health literacy among kidney disease patients, to empower those affected and their families, to make their voices heard, and engage the civil society. This year, the WKD Joint Steering Committee declares *“Kidney Health for All”* as the theme of the 2022 WKD to promote collaboration efforts between people with kidney disease, their care partners, health care providers, and all stakeholders involved to raise education and awareness on kidney health and save the lives of people with this disease.

## Conclusions

Closing the knowledge gap and improving outcomes for those with kidney disease on a global scale will require a deep understanding of the community needs. The same is true for policy development and understanding of the processes in place to engage governments worldwide, underpinned by the important principle of co-designing resources and policies that meet the needs of the community for which they are intended.

For WKD 2022, kidney organizations, including the International Society of Nephrology and International Federation of Kidney Foundations - World Kidney Alliance, should immediately advocate that the health literacy deficit is no longer a responsibility of patients but of clinicians and health policy makers. LHL occurs in all countries, regardless of income status; hence, simple, low-cost strategies are likely to be effective. Communication, universal precautions, and “teach back” can be implemented by all members of the kidney health care team. With this vision, kidney organizations will lead the shift toward improved patient-centered care, support for care partners, better health outcomes, and reduced global societal burden of kidney health care.
